# 18F-FDG PET/CT metabolism multi-parameter prediction of chemotherapy efficacy in locally progressive gastric cancer

**DOI:** 10.1007/s12149-024-01921-9

**Published:** 2024-03-27

**Authors:** Luqiang Jin, Linghe Zhang, Liping Fu, Fahuan Song, Aiping Cheng

**Affiliations:** 1 Jinzhou Medical University Postgraduate Training Base (Zhejiang Provincial People’s Hospital), Hangzhou, Zhejiang China; 2 Cancer Center, Department of Nuclear Medicine, Zhejiang Provincial People’s Hospital (Affiliated People’s Hospital), Hangzhou Medical College, 158 Shangtang Road, Hangzhou, Zhejiang China

**Keywords:** Locally progressive gastric cancer, ^18^F-FDG PET/CT, Neoadjuvant chemotherapy, Efficacy evaluation

## Abstract

**Purpose:**

This study aimed to use an ^18^F-FDG PET/CT multiparametric quantitative analysis to determine the efficacy of neoadjuvant chemotherapy in patients with locally progressive gastric cancer.

**Materials and methods:**

We conducted a retrospective analysis of 34 patients with pathologically identified gastric cancer who received neoadjuvant chemotherapy and surgery. Chemotherapy regimens were followed and ^18^F-FDG PET/CT was conducted. We ascertained multiparamaters of the target lesions pre- and post-treatment and determined the ideal cutoff values for the percentage change in biomarkers. Independent factors were evaluated using binary logistic regression. A response classification system was used to explore the association between metabolic and anatomical responses and the degree of pathological remission.

**Results:**

Binary logistic regression analysis showed that Lauren bowel type and change in total lesion glycolysis >45.2% were risk predictors for the efficacy of neoadjuvant chemotherapy; total lesion glycolysis demonstrated the best predictive efficacy. The categorical variable system of the two-module response (metabolic and anatomical response) group had a higher predictive accuracy than that of the single-module response (metabolic or anatomical response) group.

**Conclusions:**

Using ^18^F-FDG PET/CT multiparametric quantitative analysis, Lauren bowel type and change in total lesion glycolysis >45.2% were independent predictors of the efficacy of neoadjuvant chemotherapy in patients with gastric adenocarcinoma. Additionally, the dual-module assessment demonstrated high predictive efficacy.

## Introduction

In 2022, the World Health Organization ranked the incidence of gastric cancer as third and fifth most in China and worldwide, respectively, with incidence and mortality rates accounting for 10.6% and 12.5% of cancer cases in China, for 5.6% and 7.7% of all new cancers worldwide, respectively [1, 2]. Surgery up to D2 clearance remains the best treatment modality; however, the risk of recurrence remains high [3]. Previous studies have suggested that patients who receive neoadjuvant chemotherapy (NAC) demonstrate notable short-term survival advantages and considerable improvements in overall survival and prognosis [4–7]. However, NAC does not benefit all patients; some may lose the opportunity for surgery, although active treatment monitoring can determine adverse effects and avoid increased surgical complications. Moreover, the gold standard for establishing the efficacy of NAC is based on the group histopathological tumor regression grading [8], although it can only be assessed postoperatively, making it difficult to classify patients preoperatively. Therefore, a method for early and noninvasive screening of NAC efficacy has important clinical implications.

^18^F-Fluorodeoxy-glucose (^18^F-FDG) positron emission tomography (PET)/computed tomography (CT) is a commonly used molecular imaging technique that provides morphological and functional information. The prognostic value of multiparametric quantitative analyses in the evaluation of NAC efficacy and recurrence of diseases has attracted considerable attention [9–11], which assists in the development of optimal and objective individualized treatment plans.

This study aimed to evaluate the use of ^18^F-FDG PET/CT multiparametric quantitative analysis in determining the efficacy of NAC in patients with locally progressive gastric adenocarcinoma.

## Methods

### Study population

We conducted a retrospective analysis of the general, clinicopathological, and imaging data obtained from patients with locally progressive gastric adenocarcinomas who underwent surgery and NAC at our hospital between January 2019 and December 2022. We assessed patients with locally progressive gastric adenocarcinomas, which were later verified by postoperative or puncture pathologies, and who received chemotherapy regimens as advised by the National Comprehensive Cancer Network (NCCN) and the American College of Pathologists.

The inclusion criteria were as follows: (1) Contrast-enhanced CT and PET/CT scans within 1 week prior to and following neoadjuvant therapy; (2) histopathological evaluation of postoperative lesion specimens; (3) locally progressive gastric adenocarcinoma treated with chemotherapy regimens recommended by the Society of American Pathologists/NCCN guidelines, and confirmed by postoperative pathology or puncture pathology. The exclusion criteria were as follows: (1) treatment received prior to the CT and PET examinations; (2) incomplete imaging; (3) treatment refusal; (4) other known disseminated malignancies; (5) lack of usage of first-line neoadjuvant agents or combined immunotherapies, such as programmed cell death protein 1 inhibitor; (6) distant metastases detected on the initial scan; (7) missing clinical data at the time of follow-up; or 8) loss to follow-up due to treatment refusal.

### Research design

All patients were admitted to the hospital for necessary associated tests, such as magnetic resonance (MR), contrast-enhanced CT, and serological tests. Prior to NAC, baseline CT and PET/CT scans were performed. All patients then underwent more than three rounds of NAC, as recommended by the NCCN [3]. Patients were treated with fluorinated drugs, such as paclitaxel, docetaxel, fluorouracil, oxaliplatin, gemcitabine, and carbamazepine combinations. The chemotherapy schedule for the S-1 + oxaliplatin (SOX) regimen was: 80 mg/m^2^ S-1 twice per day, orally, on days 1–4 and 130 mg/m^2^ oxaliplatin intravenously on day 1, repeated every 21 days. The docetaxel + oxaliplatin + S-1 regimen was: 40 mg/m^2^ intravenous docetaxel on day 1 and 100 mg/m^2^ intravenous oxaliplatin on day 2, repeated every 21 days. The S-1 dosage was the same as previously described (oral D1–D14 twice daily, 21 days per cycle). The vinorelbine + ifosfamide + cisplatin regimen included intravenous administrations of 2 g ifosfamide on days 1–3; 30 mg/m^2^ vinorelbine on day 1; 80 mg/m^2^ cisplatin on day 1; and mesna 0, 4, 8 h after the ifosfamide, 21 days per cycle. Within 1 week of treatment completion, a second PET/CT scan was performed, and the patient underwent a thorough evaluation. Individuals who fulfilled the criteria for surgery underwent radical resection. Histological analysis of the surgical specimens was performed.

### ^18^F-FDG PET/CT scanning methods

The radiochemical purity of ^18^F-FDG was >95%, and all images were acquired using a Siemens Biograph 64 PET-CT scanner (Munich, Germany). Patients underwent a 4–6 h fast to maintain blood glucose at levels <9.1 mmol/L, received an intravenous injection of ^18^F-FDG at a rate of 5.55 MBq/kg body mass, and underwent a whole-body PET/CT scan after 50–60 min of lying still and avoiding light. During this time, patients were permitted to drink 800 mL of water to facilitate stomach dilation and bladder emptying the bladder prior to the examination. From the skull base to the midpoint of the femur, whole-body PET-CT images were obtained using a spiral CT scan with a tube voltage, current, pitch, and layer thickness of 120 kV, 170 mA, 0.75, and 0.5 mm, respectively. The ordered subset expectation maximization method was used to iteratively recreate the PET scans in three dimensions (3D) while attenuation-correcting the PET images using information from CT scans. All data were independently analyzed by two nuclear medicine physicians with over 5 years of experience in PET/CT diagnosis. The region of interest was delineated based on the lesion’s contour. The two physicians reviewed the images, engaged in discussions, and ultimately reached a consensus to determine the measurement results.

### Image processing methods

The maximum (SUVmax) and mean standard uptake values (SUVmean) of the gastric target lesion were measured automatically. The region of interest (ROI) was manually drawn using a software program (MedEX). The peak standard uptake value (SUVpeak), metabolic tumor volume (MTV), total lesion glycolysis (TLG), and lean body mass corrected standard uptake value (SUL) were measured using a 3D program (MEMRS imaging workstation) with 40% of the SUVmax as the cutoff value. The definition of the multiparameter quantitative analysis obtained from the baseline PET/CT scan was PET_1_ (SUV_1_, MTV_1_, TLG_1_, SUL_1_), and the multiparametric quantitative analysis acquired by the second PET/CT scan after the NAC treatment was PET_2_ (SUV_2_, MTV_2_, TLG_2_, SUL_2_), with ΔSUVmax% = [(SUVmax_1_ − SUVmax_2_)/SUVmax_1_]*100%. ΔMTV%, ΔTLG%, and ΔSUL% were calculated in the same way.

Contrast-enhanced CT assessment of patients undergoing NAC treatment was labeled using the Response Evaluation Criteria in Solid Tumors (RECIST) 1.1 criteria [12], which scores gastric target lesions based on the following parameters: complete remission (CR) = all target lesions disappeared and the lymph nodes were <10 mm in the short axis for at least 4 weeks; partial remission (PR) = >30% reduction in the sum of the largest longitudinal diameters of all target lesions for at least 4 weeks; disease progression (PD) = 20% increase in the sum of the largest longitudinal diameters compared with the minimum during the observation period or a ≥5 mm increase in the lesions or the appearance of new lesions; disease stabilization (SD) = total summed reduction in the target lesion diameter between CR and PD. We defined CR + PR as the anatomical imaging response group and PD + SD as the anatomical imaging non-response group. The best cutoff value calculated, based on the receiver operating characteristic (ROC) curve, was used as the boundary. The before-and-after rate of change was defined as a metabolic imaging response greater than the cutoff value and a metabolic imaging non-response lesser than the cutoff value. Changes in anatomical and metabolic imaging were jointly included in the response classification system for patient groups: two-module response (dual anatomical + metabolic response), single-module response (anatomical or metabolic response), and no-module response (anatomical + metabolic non-response) groups.

Histopathological examination of postoperative specimens and evaluation criteria.

Postoperative specimens were evaluated histopathologically. The efficacy grading system for gastric adenocarcinoma was adopted from the NCCN scoring of tumor pathological responses [13]: grade 0 (complete regression) = no tumor cell residue, including lymph nodes; grade 1 (moderate regression) = only single or small foci of cancer cell residue; grade 2 (slight regression) = tumor residue but lesser than fibrotic mesenchyme; grade 3 (no regression) = extensive tumor residue with no or little tumor cell necrosis. Patients with grades 0 and 1 were classified into the pathological response group, and patients with grades 2 and 3 were classified into the pathological non-response group.

### Statistical analysis

Statistical analyses were performed using SPSS (version 27.0; IBM Corp., Armonk, NY, USA). Quantitative variables were expressed as median and interquartile range (IQR) or mean and standard deviation. The optimal cutoff values for all PET/CT multiparameter quantitative analyses and other continuous variables were calculated using ROC curves and Jordon’s index. Parameters that were statistically significant in the one-way regression analysis were screened using stepwise linear regression analysis and included in a binary logistic regression analysis to screen for independent predictors. Comparisons of count data were performed using chi-square or Fisher’s exact tests. Differences were considered statistically significant at *P* < 0.05.

## Results

An initial cohort of 287 patients was included in the study. Of these, 34 patients who were eligible for inclusion and continuous follow-up were included in the final cohort. A summary of the patients’ baseline characteristics is presented in Table 1. The primary tumors were in the gastric cardia, corpus, and antrum. The mean duration of NAC therapy was 4 weeks (median = 4 weeks, range = 3–7 weeks). Mean SUVmax values of gastric target area lesions were 10.8 ± 6.2 (median = 9.85, range = 2.4–26.6) and 5.3 ± 3.5 (median = 5, range = 1.1–19.2) within PET_1_ and PET_2_, respectively.

**Table 1:** Patient characteristics

Characterization	Value
Age	
Mean value	62
Range	32–77
Sex	
Male	24
Female	10
CA72-4	
Median	3.75 (IQR, 1.78–15.95)
Range	0.9–287.8
CEA	
Median	3.55 (IQR, 1.58–6.28)
Range	0.7–304.3
CA19-9	
Median	15.85 (IQR, 7.23–71.70)
Range	1.8–9032
Tumor site	
Cardia of the stomach	6
Corpus	12
Antrum (anatomy)	16
Chemotherapy regimen	
SOX	17
DOS	4
NIPS	7
XELOX	6
Lauren typing	
Enteric	16
Diffuse	11
Hybrid	7
HER-2 gene	
HER-2 (0–1)	23
HER-2 (2–3)	8

*CA* cancer antigen, *CEA* carcinoembryonic antigen, *DOS* docetaxel + oxaliplatin + S-1, *HER-2* human epidermal growth factor receptor 2, *IQR* interquartile range, *NIPS* neoadjuvant intraperitoneal and systemic chemotherapy, *SOX* S-1 + oxaliplatin, *XELOX* capecitabine + oxaliplatin

**Fig. 1:**
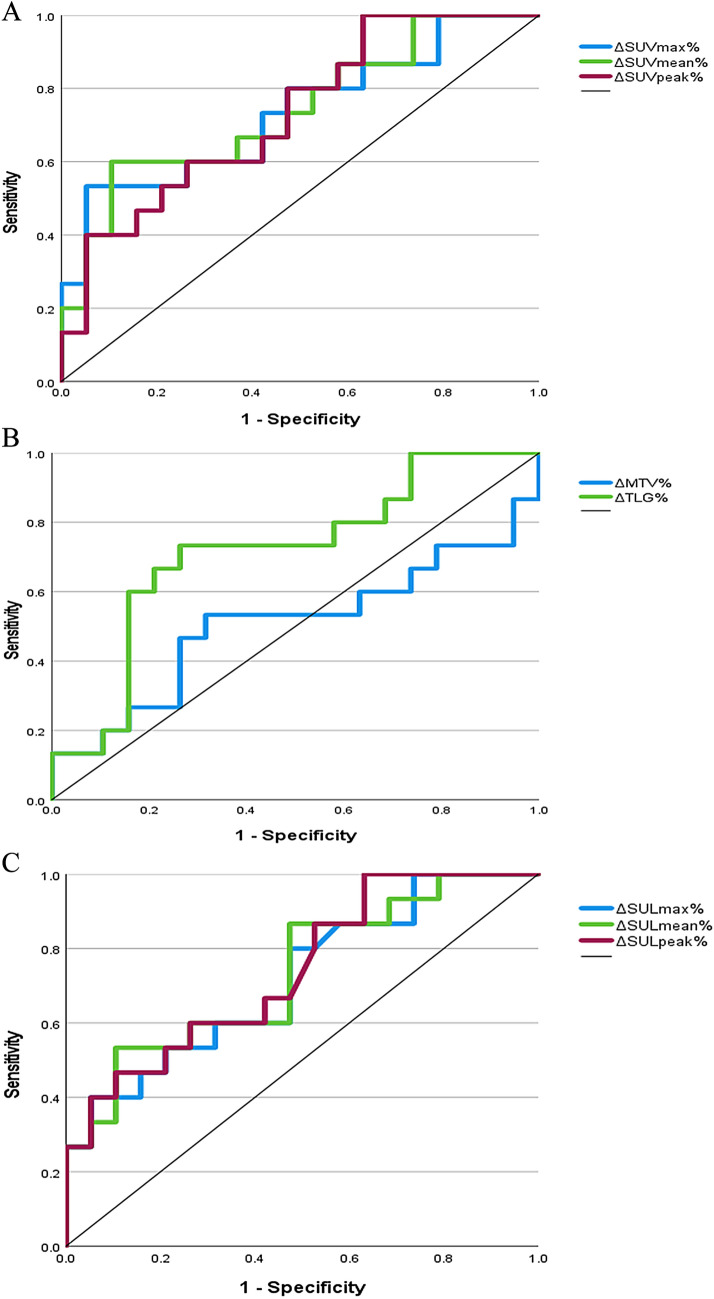
^18^F-FDG metabolic imaging biomarkers predict histopathological response receiver operating characteristic curves after neoadjuvant chemotherapy in patients with locally progressive gastric adenocarcinoma. **A** The percentage value of the reduction in SUV-related parameters between PET1 and PET2. **B** The percentage reduction in MTV and TLG between PET1 and PET2. **C** The percentage value of the reduction of SUL-related parameters between PET1 and PET2

Based on the tumor regression grading (TRG) of the histopathological response, there were 15 and 19 patients in the pathological response and non-response groups, respectively. There were no significant differences in sex, age, or tumor location, between the pathological response group and the pathological non-response group, suggesting consistent clinical features. Baseline and post-treatment ^18^F-FDG multiparameter quantitative analyses were not significantly correlated with the pathological responses (*P* > 0.05). The ROC curves (Fig. 1A–C) were analyzed to obtain the best cutoff values for ΔSUVmax, ΔSUVmean, ΔSUVpeak, ΔMTV, ΔTLG, ΔSULmax, ΔSULmean, and ΔSULpeak; values, areas under the curve (AUC), sensitivities, and specificities are presented in Table 2.

**Table 2:** Optimal cutoff values for metabolic parameters

Metabolic multiparametric	Truncation value	Sensitivity	Idiosyncrasy	AUC	*P*	95% CI
ΔSUVmax%	0.671	53.3	99.5	0.733	0.021	0.559–0.908
ΔSUVmean%	0.573	60.0	89.5	0.740	0.018	0.569–0.911
ΔSUVpeak%	0.233	100	36.8	0.730	0.023	0.561–0.899
ΔMTV%	0.088	53.3	68.4	0.505	0.959	0.295–0.716
ΔTLG%	0.452	73.3	73.7	0.716	0.033	0.537–0.895
ΔSULmax%	0.657	60.0	94.7	0.718	0.032	0.544–0.891
ΔSULmean%	0.642	53.3	89.5	0.733	0.021	0.562–0.904
ΔSULpeak%	0.234	100	36.8	0.740	0.018	0.574–0.907

*AUC* area under the curve, *CI* confidence interval, *MTV* metabolic tumor volume, *SUL* lean body mass corrected standard uptake value, *SUV* standard uptake value, *TLG* total lesion glycolysis

Lauren typing, SUVmax, SUVmean, TLG, SULmax, and SULmean were valuable in predicting the efficacy of NAC in patients with locally progressive gastric adenocarcinoma using univariate analysis. Using the pathological response as the dependent variable, clinical indicators with *P* < 0.05 after univariate analysis and ^18^F-FDG PET/CT multiparameters were included in binary logistic analysis. Lauren staging bowel type and ΔTLG% > 45.2% were independent predictors in assessing the efficacy of NAC. TLG demonstrated superior efficacy as a predictive value of the metabolic parameters (odds ratio = 5.378, 95% confidence interval 1.068–27.077, *P* = 0.041), as presented in Table 3.

**Table 3:** Single and multifactorial logistics regression analysis for predicting pathologic remission

Variables	Univariate			Multivariate		
	OR	95% CI	*P*	OR	95% CI	*P*
Age (>62 years)	1.800	0.443–7.308	0.411			
Sex (male)	0.714	0.162–3.143	0.656			
CA72-4	4.250	0.69–26.135	0.188			
CEA	1.083	0.255–4.596	0.914			
CA19-9	1.400	0.318–6.160	0.656			
Localization (sinus section)	0.450	0.111–1.827	0.264			
Surgical procedure (total radical gastrectomy)	0.923	0.218–3.916	0.914			
WHO classification (poorly differentiated carcinoma)	0.400	0.088–1.813	0.235			
RECIST 1.1 standard	19.250	2.083–177.915	0.009			
PERCIST 1.0 standard	3.792	0.655–21.961	0.137			
Lauren typing (intestinal type)	4.333	1.022–18.382	0.047	4.965	1.007–24.474	0.049
ΔSUVmax% > 67.1%	20.571	2.158–196.103	0.009			
ΔSUVmean% > 57.3%	12.750	2.123–76.572	0.005			
ΔSUVpeak% > 23.3%			–			
ΔMTV% > 8.8%	2.476	0.610–10.058	0.205			
ΔTLG% > 45.2%	4.714	1.077–20.626	0.039	5.378	1.068–27.077	0.041
ΔSULmax% > 65.7%	12.000	1.248–115.362	0.031			
ΔSULmean% > 64.2%	5.667	0.944–34.032	0.058			
ΔSULpeak% > 23.4%			–			

*CA* cancer antigen, *CEA* carcinoembryonic antigen, *CI* confidence interval, *MTV* metabolic tumor volume, *OR* odds ratio, *PERCIST* Response Criteria in Solid Tumors, *RESIST* Response Evaluation Criteria In Solid Tumors, *SUL* lean body mass corrected standard uptake value, *SUV* standard uptake value, *TLG* total lesion glycolysis, *WHO* world health organization

According to the RECIST 1.1 criteria, there were 22 patients in the anatomical imaging response group (five CR and 17 PR) and 12 patients in the anatomical imaging non-response group (nine SD and three DP); TRG and RECIST were significantly correlated with each other (*P* = 0.011). Using the 45.2% cutoff for ΔTLG, 16 and 18 patients were allocated to the metabolic imaging response and non-response groups. The dual-module response group had 15 cases and 11 pathological remissions, with 73.3% accuracy, while the single-module response group had 23 cases and 14 pathological remissions, with an accuracy of 60.9%. There was a significant difference among the dual-response, single-response, and no-response groups (*X*^2^ = 11.577, *P* = 0.02), as detailed in Table 4. The accuracy of the dual-module response group was higher than that of the single-module response group (76.5% vs. 70.6%, respectively). The peak response group, based on the PET Response Criteria in Solid Tumors (PERCIST) criteria, demonstrated the lowest accuracy, as presented in Table 5.

**Table 4:** Degree of pathological remission predicted by neoadjuvant chemotherapy between groups

Reaction classification system	Number of examples	Pathologic remission (*n* = 19)	Pathology not in remission (*n* = 15)	*X*^2^	*P*
Dual-module response group	15	11 (73.3%)	4 (26.7%)	11.577	0.02
Single-module response group	23	14 (60.9%)	9 (39.1%)		
No response group	11	1 (9.1%)	10 (90.9%)

**Table 5:** Accuracy of the response classification systems

Reaction classification system	*n*	Validity	Null	Accuracy (%)
Dual-module response group	34	26	8	76.5
Single-module response group	34	24	10	70.6
Peak response group	34	21	13	61.8
Physiology	34	34	0	100

*PERCIST* Response Criteria in Solid Tumors, *SUL* lean body mass corrected standard uptake value, *TRG* tumor regression grade

^*^ Response classification system assessed using pathologic TRG classification as the dependent variable: two-module responder group (anatomical + metabolic dual response is valid and vice versa) and single-module responder group (either anatomical or metabolic response is valid). The peak responder group refers to the PERCIST draft [19] peak response group refers to the PERCIST draft, with SULpeak = 30% as the cutoff value. Those with values greater than the cutoff value were considered as the response group

## Discussion

Active therapeutic monitoring has a pivotal role in NAC for gastric cancer, with the primary goals being assessment of tumor responsiveness to chemotherapy, determining the appropriate course of chemotherapy, and determining the optimal timing of surgery. Previous studies on PET/CT-based response assessments associated with early metabolic changes in esophagogastric junction cancer have shown significant but inconclusive results and have had an emerging role in the response to neoadjuvant treatments [14, 15]. In the present study, we assessed patients with locally progressive gastric adenocarcinomas. ^18^F-FDG PET/CT metabolic multiparameters correlated with the histopathological response to NAC and could predict chemotherapy outcomes to some extent. We also found that Lauren typing intestinal ΔTLG% > 45.2% was a risk factor for poor efficacy of NAC, which is consistent with other studies that have reported that TLG is the best marker across several cancers [16, 17]. This is mainly because quantitative parameters, such as MTV and TLG, are more responsive to systemic biology and total tumor volume than other parameters and can provide more information on the efficacy of NAC.

According to the RECIST 1.1 criteria, 26.5%, 50%, 14.7%, and 8.8% of patients demonstrated SD, PR, CR, and PD, respectively. Importantly, TRG and RECIST were significantly correlated with one another (*P* = 0.011). But it is questionable whether they can accurately reflect tumor volume in organs such as gastric cancer, which have a luminal structure and change in volume over time. In contrast, MTV is defined as the tumor volume above a certain metabolic threshold, and TLG is defined as the product of tumor volume and metabolic activity within the tumor. Meanwhile, MTV and TLG also have the additional advantage of summing multiple lesions into one representative number, this may help to further define the efficacy of evaluating patients with gastric cancer. Several studies have shown that RECIST 1.1 and PERCIST 1.0 criteria can predict the response to treatment in neoplastic diseases and that metabolic activity is more useful for efficacy classification in cases of pseudoprogression [18, 19]. Indeed, a reduction of >45.2% in quantitative TLG parameters before and after the selection of NAC improved outcome prediction compared to the 30% cutoff value, according to PERCIST. Moreover, Moore et al. [20] concluded that the currently used response criteria (i.e., PERCIST) may not be optimal, which is consistent with our results.

The categorized response system of the two-module response demonstrated higher predictive validity and necessitated further investigation. After analyzing the response classification system, the dual-module response system of functionality combined with anatomy was more accurate in predicting the efficacy of chemotherapy. The diagnostic accuracy of the two-module response was 76.5%, which could help in early and precise preoperative screening of chemotherapy-naïve patients for more aggressive treatment. Recent studies have also shown that the metabolic response is poorly correlated with the establishment of pathology (*r* = 0.121) and is not correlated with somatic changes [21]; however, they determined the cutoff values based on imaging criteria for pancreatic cancer, unlike the present study. Importantly, no standardized criteria exist for dual-module response evaluation; additional multicenter, large-scale, randomized trials are required to confirm these results. In addition, this current study can be extended to changes in diffusion-weighted MR performance, and the quantification of the apparent diffusion coefficient decay degree in combination with PET quantitative parameters has great value, and its inclusion in response classification systems requires further understanding [22]. Therefore, future guidelines should integrate new anatomical and functional metrics to develop more promising strategies.

The SOX regimen is the preferred chemotherapy regimen in Eastern countries but may not offer significantly different primary outcomes than other chemotherapy regimens [23]. In our present study, 17 patients who received an NAC SOX regimen before the gastrectomy were individually included, with a mean treatment period of 3.7 weeks. The predictive efficacy of the SOX regimen was higher than that of the other treatment regimens according to the subjects’ working curve (SUVmax, AUC 0.847 vs. 0.733). Univariate analysis showed that the ^18^F-FDG PET/CT quantitative multiparametric analysis was associated with pathological shrinkage grading, suggesting that ^18^F-FDG PET/CT metabolic multiparameters have higher value in patients using the SOX regimen, although there was no statistically significant difference.

The limitations of our current study include the relatively small cohort and the lack of multicenter cases for external validation. Future studies should validate our findings using larger, multicenter patient cohorts.

## Conclusions

The current study demonstrated that ^18^F-FDG PET/CT multiparametric quantitative analysis is valuable in assessing the efficacy of NAC for locally progressive gastric adenocarcinoma. Lauren staging intestinal ΔTLG% > 45.2% was an independent predictor for assessing the efficacy of NAC in patients with gastric adenocarcinoma, and the dual-module response assessment demonstrated high predictive efficacy. Our present results may help clinicians determine the efficacy of NAC preoperatively and provide a new tool for the personalized treatment of patients.

## Data Availability

The raw data supporting the conclusions of this article will be made available by the authors, without undue reservation.

## References

[1] Siegel RL, Miller KD, Fuchs HE, Jemal A (2022). Cancer statistics, 2022. CA Cancer J Clin.

[2] Xia C, Dong X, Li H, Cao M, Sun D, He S (2022). Cancer statistics in China and United States, 2022: profiles, trends, and determinants. Chin Med J (Engl).

[3] Ajani JA, D’Amico TA, Bentrem DJ, Chao J, Cooke D, Corvera C (2022). Gastric cancer, version 22022, NCCN clinical practice guidelines in oncology. J Natl Compr Canc Netw.

[4] Anderson E, LeVee A, Kim S, Atkins K, Guan M, Placencio-Hickok V (2021). A comparison of clinicopathologic outcomes across neoadjuvant and adjuvant treatment modalities in resectable gastric cancer. JAMA Netw Open.

[5] Lin JX, Tang YH, Lin GJ, Ma YB, Desiderio J, Li P (2022). Association of adjuvant chemotherapy with overall survival among patients with locally advanced gastric cancer after neoadjuvant chemotherapy. JAMA Netw Open.

[6] Kang YK, Yook JH, Park YK, Lee JS, Kim YW, Kim JY (2021). PRODIGY: A phase III study of neoadjuvant docetaxel, oxaliplatin, and S-1 plus surgery and adjuvant S-1 versus surgery and adjuvant S-1 for resectable advanced gastric cancer. J Clin Oncol.

[7] Su J, Guo W, Chen Z, Wang L, Liu H, Zhao L (2022). Safety and short-term outcomes of laparoscopic surgery for advanced gastric cancer after neoadjuvant immunotherapy: a retrospective cohort study. Front Immunol.

[8] Song Z, Zou S, Zhou W, Huang Y, Shao L, Yuan J (2020). Clinically applicable histopathological diagnosis system for gastric cancer detection using deep learning. Nat Commun.

[9] Abdelrahman AM, Goenka AH, Alva-Ruiz R, Yonkus JA, Leiting JL, Graham RP (2022). FDG-PET predicts neoadjuvant therapy response and survival in borderline resectable/locally advanced pancreatic adenocarcinoma. J Natl Compr Canc Netw.

[10] van der Hiel B, Blankenstein SA, Aalbersberg EA, Wondergem M, Stokkel MPM, van de Wiel BA (2022). ^18^F-FDG PET/CT during neoadjuvant targeted therapy in prior unresectable stage III melanoma patients: can (early) metabolic imaging predict histopathologic response or recurrence?. Clin Nucl Med.

[11] Lim CH, Park YJ, Shin M, Cho YS, Choi JY, Lee KH (2020). Tumor SUVs on ^18^F-FDG PET/CT and aggressive pathological features in esophageal squamous cell carcinoma. Clin Nucl Med.

[12] Eisenhauer EA, Therasse P, Bogaerts J, Schwartz LH, Sargent D, Ford R (2009). New response evaluation criteria in solid tumours: revised RECIST guideline (version 1.1). Eur J Cancer.

[13] Hall WA, Li J, You YN, Gollub MJ, Grajo JR, Rosen M (2023). Prospective correlation of magnetic resonance tumor regression grade with pathologic outcomes in total neoadjuvant therapy for rectal adenocarcinoma. J Clin Oncol.

[14] Sánchez-Izquierdo N, Perlaza P, Pagès M, Buxó E, Rios J, Rubello D (2020). Assessment of response to neoadjuvant chemoradiotherapy by ^18^F-FDG PET/CT in patients with locally advanced esophagogastric junction adenocarcinoma. Clin Nucl Med.

[15] Moore JL, Subesinghe M, Santaolalla A, Green M, Deere H, Van Hemelrijck M (2023). Metabolic tumour and nodal response to neoadjuvant chemotherapy on FDG PET-CT as a predictor of pathological response and survival in patients with oesophageal adenocarcinoma. Eur Radiol.

[16] Nose Y, Makino T, Tatsumi M, Tanaka K, Yamashita K, Noma T (2023). Risk stratification of oesophageal squamous cell carcinoma using change in total lesion glycolysis and number of PET-positive lymph nodes. Br J Cancer.

[17] Güç ZG, Turgut B, Avci A, Cengiz F, Eren Kalender M, Alacacioğlu A (2022). Predicting pathological response and overall survival in locally advanced gastric cancer patients undergoing neoadjuvant chemotherapy: the role of PET/computed tomography. Nucl Med Commun.

[18] Beer L, Hochmair M, Haug AR, Schwabel B, Kifjak D, Wadsak W (2019). Comparison of RECIST, iRECIST, and PERCIST for the evaluation of response to PD-1/PD-L1 blockade therapy in patients with non-small cell lung cancer. Clin Nucl Med.

[19] Velez EM, Desai B, Ji L, Quinn DI, Colletti PM, Jadvar H (2020). Comparative prognostic implication of treatment response assessments in mCRPC: PERCIST 1.0, RECIST 1.1, and PSA response criteria. Theranostics..

[20] Benz MR, Armstrong WR, Ceci F, Polverari G, Donahue TR, Wainberg ZA (2022). ^18^F-FDG PET/CT Imaging biomarkers for early and late evaluation of response to first-line chemotherapy in patients with pancreatic ductal adenocarcinoma. J Nucl Med.

[21] Dalah E, Tai A, Oshima K, Hall WA, Erickson B, Li XA (2018). PET-based treatment response assessment for neoadjuvant chemoradiation in pancreatic adenocarcinoma: an exploratory study. Transl Oncol.

[22] Tang L, Wang XJ, Baba H, Giganti F (2020). Gastric cancer and image-derived quantitative parameters: Part 2-a critical review of DCE-MRI and ^18^F-FDG PET/CT findings. Eur Radiol.

[23] Sah BK, Zhang B, Zhang H, Li J, Yuan F, Ma T (2020). Neoadjuvant FLOT versus SOX phase II randomized clinical trial for patients with locally advanced gastric cancer. Nat Commun.

